# Distributed Synchronization Technique for OFDMA-Based Wireless Mesh Networks Using a Bio-Inspired Algorithm

**DOI:** 10.3390/s150818287

**Published:** 2015-07-28

**Authors:** Mi Jeong Kim, Sung Joon Maeng, Yong Soo Cho

**Affiliations:** School of Electrical and Electronic Engineering, Chung-Ang University, Seoul 156-756, Korea; E-Mails: kimmj@cau.ac.kr (M.J.K); aod0527@naver.com (S.J.M)

**Keywords:** wireless mesh network, OFDMA, bio-inspired algorithm, distributed synchronization, time difference of arrival, time synchronization, frequency synchronization

## Abstract

In this paper, a distributed synchronization technique based on a bio-inspired algorithm is proposed for an orthogonal frequency division multiple access (OFDMA)-based wireless mesh network (WMN) with a time difference of arrival. The proposed time- and frequency-synchronization technique uses only the signals received from the neighbor nodes, by considering the effect of the propagation delay between the nodes. It achieves a fast synchronization with a relatively low computational complexity because it is operated in a distributed manner, not requiring any feedback channel for the compensation of the propagation delays. In addition, a self-organization scheme that can be effectively used to construct 1-hop neighbor nodes is proposed for an OFDMA-based WMN with a large number of nodes. The performance of the proposed technique is evaluated with regard to the convergence property and synchronization success probability using a computer simulation.

## 1. Introduction

Wireless mesh networks (WMNs) based on orthogonal frequency division multiple access (OFDMA) have been viewed with considerable interest because they can increase the data rates and flexibility of resource allocation while avoiding the interference between multichannels [1]. OFDMA-based WMNs have been investigated for various applications, such as enterprise networking, wireless networks for public safety, and tactical information and communication networks [2]. One of the prominent challenges in distributed OFDMA-based WMNs is the synchronization between nodes, particularly when a global positioning system (GPS) signal is not available for a reference timing. A ranging procedure between the base station and mobile station resolves the synchronization issue in centralized networks such as 3G, LTE, and WiMAX systems. However, because the nodes in a distributed WMN can directly communicate with each other without a central controller, each node receives signals from the neighbor nodes with a time difference of arrival (TDoA), resulting in intersymbol interference (ISI) and intercarrier interference (ICI). Unlike cellular networks, the network topology in a distributed WMN can change frequently.

In [3], closed-form expressions for the average signal-to-interference ratio at the fast Fourier transform (FFT) output in the presence of timing offsets and carrier-frequency offsets (CFOs) among the users in an uplink OFDMA are derived. Additionally, a parallel interference canceller (PIC) is derived in order to mitigate the effects of the timing offsets and CFOs by using a matched filter detector in the case of a binary phase-shift keying modulation. In [4], a multistage linear PIC approach is proposed to mitigate the effects of multiuser interferences due to CFOs in an uplink OFDMA. In [5], an interference-mitigation technique based on the extension of the cyclic prefix (CP) and dynamic positioning of the FFT windows is developed for OFDMA wireless ad-hoc networks with arbitrary timing misalignments. In [6], a distributed clock-synchronization technique using the group neighborhood average is proposed for wireless sensor networks. Here, the group averages of the offset and skew rate, measured at each sensor node, are used for the global synchronization. This technique allows the sensor network to quickly establish a consensus clock and maintain a small deviation from it.

Recently, several distributed synchronization techniques were developed for OFDMA-based WMNs with TDoAs. In [7], a timing-adjustment technique for mitigating the interference between multiple nodes in an asynchronous OFDMA mesh network using a non-iterative algorithm is proposed. In [8], a successive detection technique is proposed to mitigate the effects of the interferences caused by TDoAs in an OFDMA-based WMN. However, its computational complexity and latency are relatively high, as filtering operations for the forward and backward cancellations in a frame are needed to remove the ISI and ICI caused by the TDoAs. In [9,10], a distributed time- and frequency-synchronization technique for OFDMA-based WMNs is proposed by updating the FFT starting points, transmission times, and carrier frequencies according to the request messages received from the neighbor nodes.

Bio-inspired algorithms, which explore the behavioral principles of living organisms, have been successfully applied in various fields, including communication networks [11]. A principle regarding the colonial movement of living organisms suggests that an entire organism autonomously maintains a pattern of behavior without the help of a central leader. Each individual adapts its movement to the local environment in a distributed manner, yielding the synchronization of the entire colonial movement [12,13,14]. The term “distributed” implies that the adjustment of the individual behavior is purely local with simple rules and not controlled by a single leader (or a few leaders). In this paper, distributed time- and frequency-synchronization techniques based on a bio-inspired algorithm are proposed for nodes in an OFDMA-based WMN with a TDoA. Updating formulas for the distributed synchronization are proposed by considering the propagation delay between the nodes, which is essential in real wireless communication channels. The proposed techniques, which are based on a flocking algorithm, achieve distributed synchronization using only the received signals from the neighbor nodes (without requiring feedback channels) and a simple algorithm. Furthermore, a self-organization scheme that can effectively construct 1-hop neighbor nodes is proposed for an OFDMA-based WMN with a large number of nodes.

The paper is organized as follows: a preliminary discussion of an OFDMA-based WMN with a TDoA and the flocking algorithm are presented in Section 2. In Section 3, a distributed time- and frequency-synchronization technique based on the flocking algorithm is proposed. The performance of the proposed technique is evaluated using computer simulations in Section 4. Finally, conclusions are drawn in Section 5.

## 2. Preliminaries

In this paper, we consider an OFDMA-based WMN without a cluster head. To avoid co-channel interference among the distributed nodes, we assume that all the resources in the 1-hop neighbor nodes are allocated orthogonally in the frequency domain. We are primarily concerned with the initialization stage of the network synchronization, which occurs prior to the data transmission. For the i th node in an OFDMA-based WMN, the received signal $y_{i}(t)$ can be represented as:

(1) $$y_{i}(t)=\sum_{j=0}^{N_{i}}\sum_{k=0}^{K-1}H_{ij}[k]X_{j}[k]\exp(j2\pi k\text{Δ}f(t-\tau_{ij}))+z_{i}(t)$$

where $H_{ij}[k]$ is the channel transfer function between the node i and the neighbor node j , and $X_{j}[k]$ is the OFDMA signal transmitted from the 1-hop neighbor node j . $N_{i}$ ,  Δf , K , $\tau_{ij}$ , and $z_{i}(t)$ denote the number of 1-hop neighbor nodes surrounding the node i , subcarrier spacing, number of allocated subcarriers, propagation delay between the nodes i and j , and noise, respectively.

The arrival time of a signal transmitted from a neighbor node, sampled with a period of $T_{s}$ , can be represented by considering the propagation delay between the nodes, as follows:

(2) $$t_{j\to i}(n)=t_{j}(n)+\tau_{ij}\text{, }j=1,\cdots ,N_{i}$$

where $t_{j\to i}(n)$ denotes the arrival time of the signal from the neighbor node j at the node i , and $t_{j}(n)$ denotes the transmission time of the node j in the n th sampling period.

**Figure 1:**
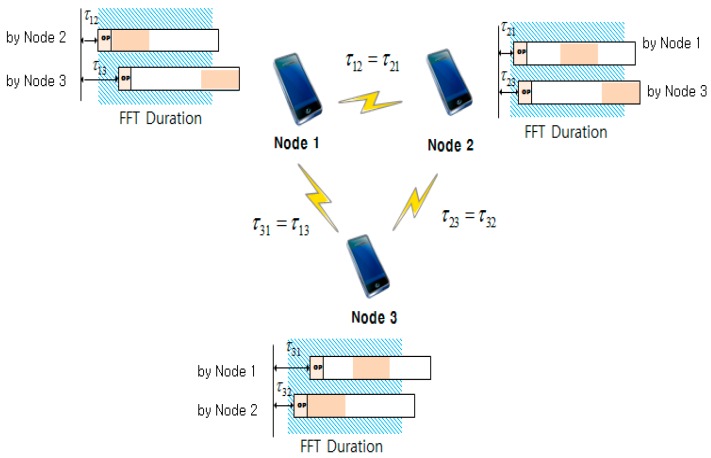
Example of an OFDMA-based WMN ( N=3 ).

Figure 1 shows an example of an OFDMA-based WMN consisting of three mesh nodes with different propagation delays. If Figure 1 is operated as a centralized network wherein Node 1 is a control node, each client node (Nodes 2 and 3) sends a ranging symbol to the control node (Node 1) at the start of the ranging process. Upon receiving the ranging symbol, the control node estimates the round-trip propagation delay using the symbol timing offset (STO) estimation technique. Then, it sends back to each client node a ranging response indicating the value of the timing advance (TA). Finally, each client node transmits a data burst after precompensating it with the TA information received from the control node. However, the synchronization between the client nodes (Nodes 2 and 3) cannot be achieved in this network. Because each node in WMNs can directly communicate with other nodes without a central controller, there can be ISI and ICI in the received signal. If Figure 1 is operated as a distributed WMN, any node can communicate directly with the neighbor nodes. For instance, Node 1 can receive signals simultaneously from Nodes 2 and 3 in OFDMA-based WMNs. At Node 1, the TDoA of the signals from Nodes 2 and 3 is given as:

(3) $$TDoA_{1}(n)=\left|\left(t_{2}+\tau_{12})-(t_{3}+\tau_{13}\right)\right|$$

A TDoA may occur because of different propagation delays even when all the nodes in the WMN are synchronized (identical transmission times). In an OFDMA-based WMN, such interferences caused by the neighbor nodes with a TDoA result in a loss of orthogonality, significantly degrading the performance even with orthogonal resource allocation. A simple technique for mitigating the interferences caused by the TDoA in an OFDMA-based WMN is to use an extended CP that is larger than the sum of the maximum TDoA and maximum excess channel delay. However, this decreases the spectral efficiency, as the extended CP should be inserted in every OFDM symbol for all nodes.

Although the extended CP can be used for a large coverage, it is always preferable that the estimated starting point of the OFDM symbol is precisely or slightly earlier than the exact timing instance. If the estimated starting point of the OFDM symbol is before the exact point but after the end of the (lagged) channel response to the previous OFDM symbol, the symbol does not overlap with the previous OFDM symbol and does not incur any ISI due to the previous symbol. In this case, the phase offset caused by the STO can be easily compensated by a single-tap frequency-domain equalizer. However, if the estimated starting point of the OFDM symbol is too much earlier than the exact timing instance (*i.e.*, the estimated starting point of the OFDM symbol is prior to the end of the (lagged) channel response to the previous OFDM symbol), the orthogonality among the subcarrier components is destroyed by the previous symbol, yielding ISI and ICI. Therefore, it is always better to synchronize the symbol timing as closely as possible with the exact timing instance or make it slightly earlier than the exact timing instance.

Bio-inspired algorithms such as ant-colony, bee, and flocking algorithms have been applied to communication networks owing to their useful characteristics. In particular, they are adaptable to various environmental conditions, inherently resilient to failure and damage, and able to perform collaborative operations based on a limited set of rules. In this paper, a flocking algorithm is used for time and frequency synchronization in an OFDMA-based WMN with a TDoA. The term “flocking” represents the phenomenon whereby self-propelled individuals are organized into an ordered motion using limited environmental information and simple rules. In [14], F. Cucker and S. Smale provide a mathematical model describing the evolution of a flock. The Cucker-Smale model (C-S model) is given as follows:

(4) $$\frac{d}{dt}x_{i}(t)=v_{i}(t)\;t>0,i=1,\cdots ,N$$

(5) $$\frac{d}{dt}v_{i}(t)=\frac{\lambda }{N}\sum_{j=1}^{N}\psi \left(\left|x_{j}(t)-x_{i}(t)\right|\right)\left(v_{j}(t)-v_{i}(t)\right)$$

where $x_{i}(t)$ and $v_{i}(t)$ are the location and velocity, respectively, of the i th agent. The variables N , λ , and Ψ(⋅) denote the number of agents, coupling strength (learning factor), and communication-range function, respectively. In the C-S model, each self-propelled agent adjusts its velocity according to the velocities of its neighbors. As t→∞ , the N agents exhibit time-asymptotic flocking phenomena. The velocities of the N agents converge to the average velocity for all the agents.

## 3. Distributed Time- and Frequency-Synchronization Techniques for an OFDMA-Based WMN

The proposed time- and frequency-synchronization techniques for an OFDMA-based WMN with a TDoA are based on the C-S model, wherein each individual locally adapts its movement to the surrounding environment in a distributive manner. If an individual’s initial velocity and position are within a certain range, the convergence of the recursive algorithm is guaranteed according to the C-S model [13,14,15]. By considering the agent and its velocity in the C-S model as a node and its transmission time in a WMN, a modified version of the C-S model (modified C-S model) for distributed time-synchronization can be described in a discrete-time domain as follows:

(6) $$t_{i}(n+1)-t_{i}(n)=\frac{\lambda }{N_{i}}\sum_{j=1}^{N_{i}}\left(t_{j}(n)-t_{i}(n)\right)$$

In Equations (4) and (5), it is assumed that the neighbors’ velocities can be detected instantly by observing the movements of neighboring individuals. In real wireless communication channels, unlike the C-S model, there is always a propagation delay between nodes i and j . Therefore, the transmission times of the neighbor nodes in the modified C-S model should be changed to the arrival times by considering the propagation delay between the nodes. The transmission time of each node in a practical WMN should be updated using the arrival time of the signal from the neighbor node j at the node i , as follows:

(7) $$\begin{array}{l} t_{i}(n+1)-t_{i}(n)=\frac{\lambda }{N_{i}}\sum_{j=1}^{N_{i}}\left(t_{j\to i}(n)-t_{i}(n)\right)\\ \text{​​​​​ }=\frac{\lambda }{N_{i}}\sum_{j=1}^{N_{i}}(t_{j}(n)-t_{i}(n))+\frac{\lambda }{N_{i}}\sum_{j=1}^{N_{i}}\tau_{ij} \end{array}$$

In Equation (7), the error term given by the average of the propagation delays is accumulated in the distributed time-synchronization process. To use the modified C-S model in a practical situation, the estimation and compensation of the propagation delays for all the neighbor nodes are needed; that is, each node must estimate the propagation delays from its neighbor nodes and request TAs to its neighbor nodes through feedback channels. This precompensation technique has been applied in centralized cellular systems by using an initial ranging signal or periodic ranging signal. However, it is inadequate for distributed WMNs wherein the mesh topology may frequently change. It also requires a large amount of resources, such as time, bandwidth, and network delay, because of the frequent requests through the feedback channels. In this paper, a distributed time-synchronization technique for achieving fast synchronization without a feedback channel in an OFDMA-based WMN with a TDoA is described.

**Figure 2:**
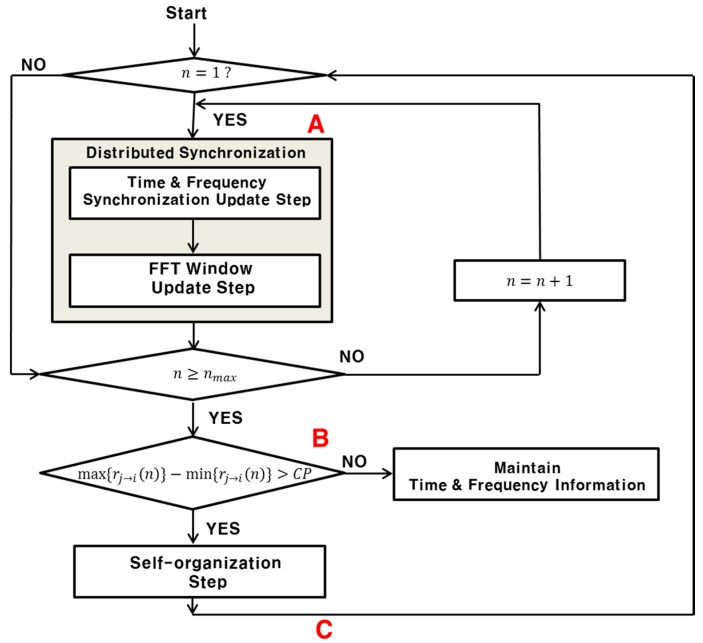
Flowchart of the proposed distributed synchronization technique.

Figure 2 shows a flowchart of the proposed distributed time- and frequency-synchronization techniques. The proposed synchronization techniques comprise three steps: the time and frequency update (transmit mode), FFT window update (receive mode), and self-organization. In the time and frequency update, the transmission time and frequency for each node are updated using the signals received from the neighbor nodes. The main focus of the proposed technique lies in this step, where the bio-inspired algorithm is used to achieve the time and frequency synchronization in a distributed manner. The formulas for updating the transmission time and frequency for the node i are given as:

(8) $$t_{i}(n+1)=c_{t}\cdot t_{i}(n)+\mu_{t}\cdot \text{Δ}t_{i}(n)$$

(9) $$f_{i}(n+1)=c_{f}\cdot f_{i}(n)+\mu_{f}\cdot \text{Δ}f_{i}(n)$$

In Equations (8) and (9), each node i updates the next transmit time $t_{i}(n+1)$ and frequency $f_{i}(n+1)$ using the arrival time $t_{j\to i}(n)$ and frequency $f_{j}(n)$ from a neighbor node j . Parameters ( $c_{t}$ , $c_{f}$ , $\mu_{t}$ , $\mu_{f}$ ) control the convergence speed and error accumulation caused by the propagation delays in the time and frequency update step. $\text{Δ}t_{i}(n)$ and $\text{Δ}f_{i}(n)$ denote the timing and frequency correction terms comprising the average timing and frequency errors, respectively, between the node i and its neighbor nodes:

(10) $$\text{Δ}t_{i}(n)=\sum_{j=1}^{N_{i}}\alpha_{ij}(t_{j\to i}(n)-t_{i}(n))$$

(11) $$\text{Δ}f_{i}(n)=\sum_{j=1}^{N_{i}}\alpha_{ij}(f_{j}(n)-f_{i}(n))$$

The term ${\text{α}}_{ij}$ denotes the weighting factor between the nodes i and j , and it is set as $\sum_{j}{\text{α}}_{ij}=1$ . The initial weight ${\text{α}}_{ij}$ is set as $1/N_{i}$ . According to Equations (10) and (11), a feedback channel is not required, because the arrival time and frequency at each node can be estimated using only the received signal. In the proposed technique, it is assumed that each node periodically transmits a preamble prior to the data transmission. Then, the node i can estimate the arrival time $t_{j\to i}(n)$ and frequency $f_{j}(n)$ according to the preambles received from the neighbor nodes. The effect of the propagation delay on the time-updating formula is indicated by the following equation:

(12) $$\begin{array}{l} t_{i}(n+1)=c_{t}\cdot t_{i}(n)+\mu_{t}\cdot \frac{1}{N_{i}}\sum_{j=1}^{N_{i}}\left(t_{j\to i}(n)-t_{i}(n)\right)\\ \;=\underset{conventional\;Flocking\;model}{\underset{︸}{c_{t}\cdot t_{i}{\left(n\right)}_{t}+\mu_{t}\cdot \frac{1}{N_{i}}\sum_{j=1}^{N_{i}}\left(t_{j}(n)-t_{i}(n)\right)}}\;+\underset{\text{error term caused by propagation delay}}{\underset{︸}{\mu_{t}\cdot \frac{1}{N_{i}}\sum_{j=1}^{N_{i}}\tau_{ij}}} \end{array}$$

When there is no propagation delay between the nodes, the formula with the parameters $c_{t}=\mu_{t}=1$ becomes the modified C-S model (discrete-time version of C-S model). However, the convergence property is not guaranteed for the C-S model when there is a propagation delay. The appropriate values of the synchronization parameters, $c_{t}$ and $\mu_{t}$ , must be determined for the convergence of the updating formula. To analyze the convergence property of the proposed technique, the transfer function of the updating formula is expressed in the *z*-domain. If we regard the arrival time of the signal from a neighbor node as the input and the transmission time of the node i as the output, the transfer function of the updating formula for the node i in Equations (8) and (9) can be derived in the *z*-domain as:

(13) $$H_{i}(z)=\frac{\mu_{t}}{z-c_{t}+\mu_{t}}$$

Note that $H_{i}(z)$ has a real pole at $c_{t}-\mu_{t}$ . If this pole lies within the unit circle in the *z*-plane, the proposed updating formula are always asymptotically stable. To reduce the accumulated error term $\frac{\mu_{t}}{N_{i}}\sum_{j=1}^{N_{i}}\tau_{ij}$ caused by the propagation delays in the transmit-time updating step of Equation (12), $\mu_{t}$ should be selected such that $\mu_{t}<0$ . Thus, in order to satisfy the two convergence conditions for the time synchronization, $c_{t}$ and $\mu_{t}$ should be selected such that:

(14) $$\mathrm{and}-1<c_{t}-\mu_{t}<1$$

If $\mu_{t}=c_{t}=-1$ , the proposed updating formula operates in a zero-pole condition that exhibits the fastest convergence speed. If $\mu_{t}=-1$ , the accumulated error is minimized, yielding the minimal TDoA after the convergence. The convergence condition for the frequency synchronization can be derived in the same way as that for the time synchronization. Unlike the time synchronization, the frequency synchronization is not affected by the propagation delay in wireless channels. Thus, the convergence condition for the proposed frequency-synchronization technique is given as:

(15) $$-1<c_{f}-\mu_{f}<1$$

In the FFT window update step, the starting point of the FFT window is updated with the signals received from the neighbor nodes. The updating formula for the FFT window position of the node i is given as:

(16) $$FFT_{i}(n+1)=\min\left(t_{j\to i}(n)\right)+T_{CP},\forall \;j$$

where $T_{CP}$ is the CP duration. The starting point of the FFT window in the receive mode is determined by the first arrival time among the signals transmitted from the neighbor node. When the maximum TDoA is smaller than the CP duration, the interference caused by the different signals’ arrival times can be ignored by selecting the FFT window position according to Equation (16). By iterating the updating formulas of Equations (8) and (10), the proposed time-synchronization technique can achieve a global synchronization without any additional precompensation procedures. Under the ideal conditions, the different transmission times of all the nodes converge to a common average value as in the C-S model. However, a TDoA may exist after the convergence, especially when a 1-hop neighbor node is located far away with a large propagation delay. The maximum bound for the TDoA of each node after the convergence is determined by the difference between the maximum and minimum propagation delays, as follows:

(17) $$0\;\leq \;TDoA_{i}\;\leq \;\left|\max(\tau_{ij})-\min(\tau_{ij})\right|\text{, }\forall \;j$$

It is possible to reach the maximum bound of the TDoA in Equation (15) when the transmission times of all the nodes are synchronized using a GPS. In this case, the arrival time from a neighbor node is determined only by the propagation delay between the nodes; hence, the maximum bound of the TDoA is given by the right-hand side of Equation (17). When a 1-hop neighbor node is located far away, the maximum TDoA after the convergence may be larger than the CP duration. In this case, the WMN violates the convergence condition (all TDoAs from 1-hop neighbor nodes are within the CP duration), causing a loss of orthogonality. The WMN can then be reorganized (self-organization) by updating the weights in Equation (10) as follows:

(18) $$\alpha_{ij}(n+1)=\{\begin{array}{c} \alpha_{ij}(n)\\ \;\\ 0 \end{array}\begin{array}{c} \text{ if }\min(t_{j\to i}(n))\leq t_{j\to i}(n)\;\\ \;<\max(t_{j\to i}(n))+T_{CP})\;\\ \text{otherwise} \end{array}\;$$

The weight between the nodes i and j is maintained if the arrival time of the signal from the node j falls within the CP duration. Otherwise, the weight ${\text{α}}_{\text{ij}}$ is set as 0, implying that the 1-hop link between the nodes i and j is physically disconnected. In this case, the 1-hop neighbor list must be reconfigured through a re-routing procedure so that the disconnected node can be supported by its neighbor node. The performance loss caused by disconnecting the node is insignificant because the link with a large propagation delay usually experiences a significant path loss. Table 1 compares the proposed distributed synchronization technique with three different previous techniques developed for OFDMA-based WMSs [7,8,9,10].

**Table 1:** Comparison of distributed synchronization techniques for OFDMA-based WMSs.

	Reference [10]	Reference [9]	Reference [7]	Proposed
Iterative/non-iterative	Iterative	Iterative	Non-iterative	Iterative
Consensus algorithm	Yes	Yes	-	-
Flocking algorithm	-	-	-	Yes
Feedback channel	Required	Required	Required	Not required
Computational complexity	Medium	Low	High	Low

## 4. Simulation Results

In this section, the performance of the proposed distributed time-synchronization technique for an OFDMA-based WMN with TDoAs is evaluated by a simulation. The parameters used for the simulation are taken from the profiles in the IEEE 802.16e standard (mobile WiMAX) [16,17]. RMa LoS is used for the channel model [18]. The locations of the nodes are randomly chosen. The specific values of the parameters used in the simulation are as follows: (a) the size of the FFT/IFFT( $N_{FFT}$ ) is 2048; (b) the CP length is 512; (c) the sampling period ( $T_{s}$ ) is 0.129 us/sample; (d) the number of nodes in a WMN ( N ) is 3, 4, and 10; (e) the maximum number of 1-hop nodes ( $N_{i}$ ) is 1/(N−1) ; and (f) the initial weights ( ${\text{α}}_{ij}$ ) are set as $1/N_{i}$ . A total of 1000 simulation runs are conducted for all the scenarios in this section.

**Figure 3:**
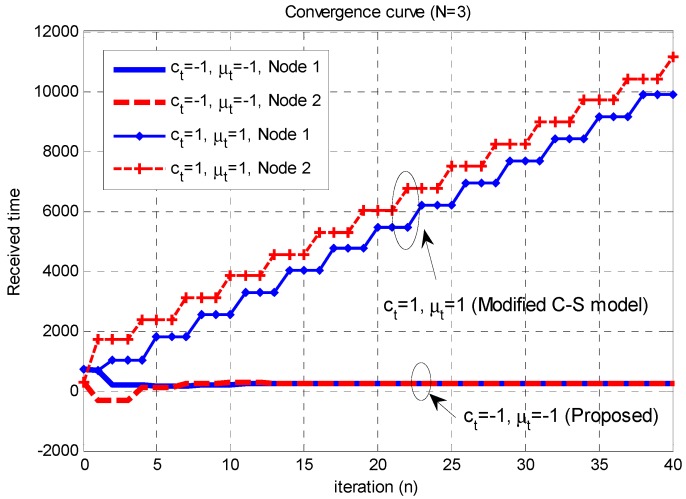
Convergence curves of the modified C-S model and proposed time-synchronization technique with the propagation delays ( $\left[\tau_{12},\;\tau_{23},\;\tau_{13}]=[394,320,330\right]$ , $\left[t_{1},\;t_{2},\;t_{3}]=[918,-52,247\right]$ ).

Figure 3 compares the performance of the conventional flocking model and proposed time-synchronization technique when the propagation delays are present. In this figure, the *y*-axis represents the arrival times of the signals from the neighbor Nodes 1 and 2 at the Node 3 when N=3 . Here, $\left[t_{1},\;t_{2},\;t_{3}\right]$ denotes the transmission times of each node, and $\left[\tau_{12},\;\tau_{23},\;\tau_{13}\right]$ denotes the propagation delays between the nodes. As discussed in Section 3, the modified C-S model is the special case of the proposed time-synchronization algorithm for $c_{t}=\mu_{t}=1$ . Because the modified C-S model (conventional flocking algorithm) does not satisfy the second requirement ( μ<0 ) for the convergence, the received times in this figure do not converge, owing to the error accumulation. On the other hand, when the convergence conditions are satisfied ( $c_{t}=-1,\;\mu_{t}=-1$ ), the TDoA after the convergence is smaller than the CP duration.

**Figure 4:**
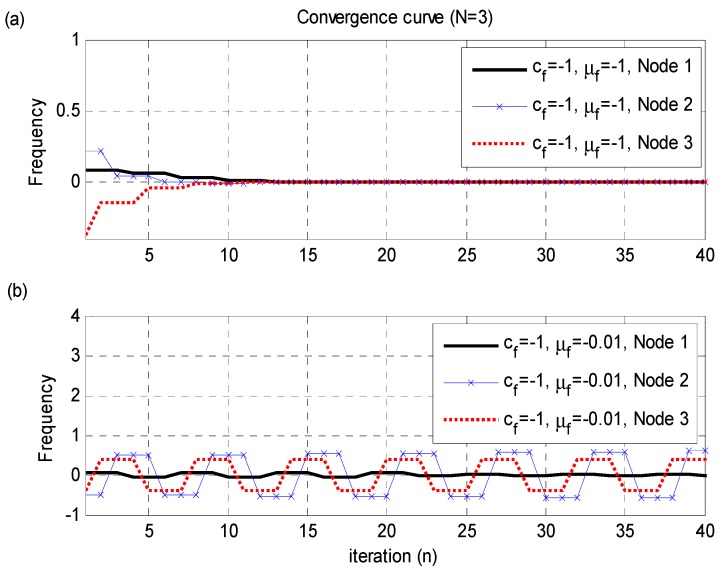
Convergence curve of the proposed frequency-synchronization technique when the propagation delays ( $\left[\tau_{12},\;\tau_{23},\;\tau_{13}]=[394,320,330\right]$ , $\left[t_{1},\;t_{2},\;t_{3}]=[918,-52,247\right]$ ) are present.

**Figure 5:**
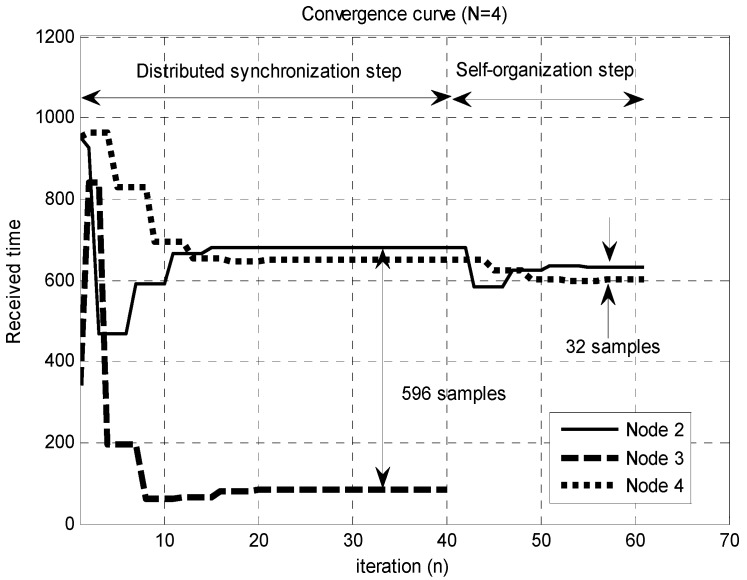
Convergence curve of the proposed synchronization technique when the propagation delays ( $\left[\tau_{12},\;\tau_{23},\;\tau_{13},\tau_{14},\tau_{24},\tau_{34}]\;=[951,342,952,709,186,769\right]$ , $\left[t_{1},\;t_{2},\;t_{3},\;t_{4}]=[150,-25,500,10\right]$ ) are present.

Figure 4 shows a convergence curve of the proposed frequency-synchronization technique when the propagation delays are present. Figure 4a shows the case ( $c_{f}=-1$ , $\mu_{f}=-1$ ) where the parameters $c_{f}$ and $\mu_{f}$ satisfy the condition. Figure 4b shows the case ( $c_{f}=-1$ , $\mu_{f}=0.01$ or $c_{f}-\mu_{f}=-1.01$ ) where the parameters $c_{f}$ and $\mu_{f}$ do not satisfy the condition. In this figure, we observe that the parameters satisfying the condition in Equation (15) should be selected for the convergence of the frequency-synchronization technique in Equation (9). Figure 5 shows a convergence curve of the proposed distributed time-synchronization technique for N=4 . Here, the maximum TDoA (596 samples) after the convergence is larger than the CP duration (512 samples). Because this violates the convergence condition, the self-organization step is applied, reducing the TDoA to 32 samples at completion.

Table 2 summarizes the synchronization success probability of the proposed technique for N=3,  4 , and 10. Here, it is assumed that “synchronization success” is achieved when each node in a WMN is synchronized not only with the target node but also with all the 1-hop neighbor nodes, with TDoA being smaller than the CP duration. In Table 2, “Before distributed synchronization”, “Only distributed synchronization”, and “Synchronization and self-organization” refer to the synchronization success probabilities at the points A, B, and C shown in Figure 2, respectively. Case I corresponds to the case where the node with the largest TDoA is disconnected. Case II corresponds to the case where all the 1-hop nodes not satisfying the convergence condition are disconnected.

**Table 2:** Synchronization success probability of the proposed technique.

Step		N=3	N=4	N=10
Before distributed synchronization		34%	27%	0%
Only distributed synchronization		100%	99%	78%
Synchronization and self-organization	Case I	-	100%	88%
	Case II	-	-	100%

In Table 2, we observe that the synchronization success can be achieved with only the distributed synchronization in most cases when N is small (3 or 4). Figure 5 ( N=4 ) corresponds to the worst case (1%), where the synchronization success cannot be achieved with only the distributed synchronization. As N increases, the synchronization success probability decreases because of the large number of neighbor nodes that must be synchronized. For N=10 , the synchronization success probability obtained by only the distributed synchronization is 78%. The self-organization step improves the success probability to 88 and 100% in Cases I and II, respectively. The average number of disconnected nodes in Case II is 2.6.

**Figure 6:**
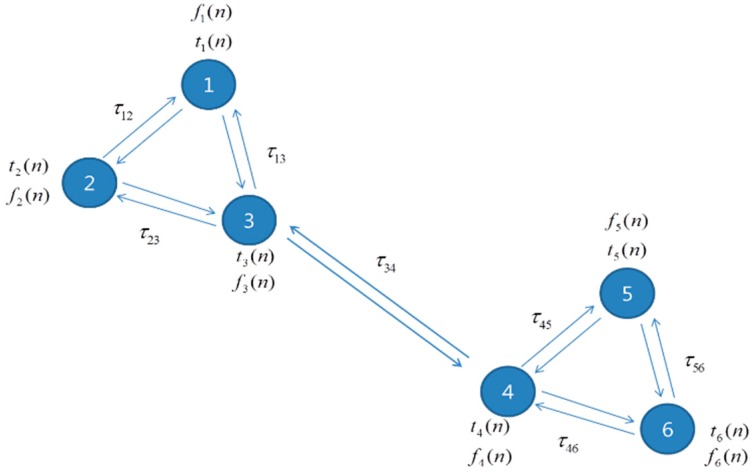
Multi-hop network topology used in the simulation.

**Figure 7:**
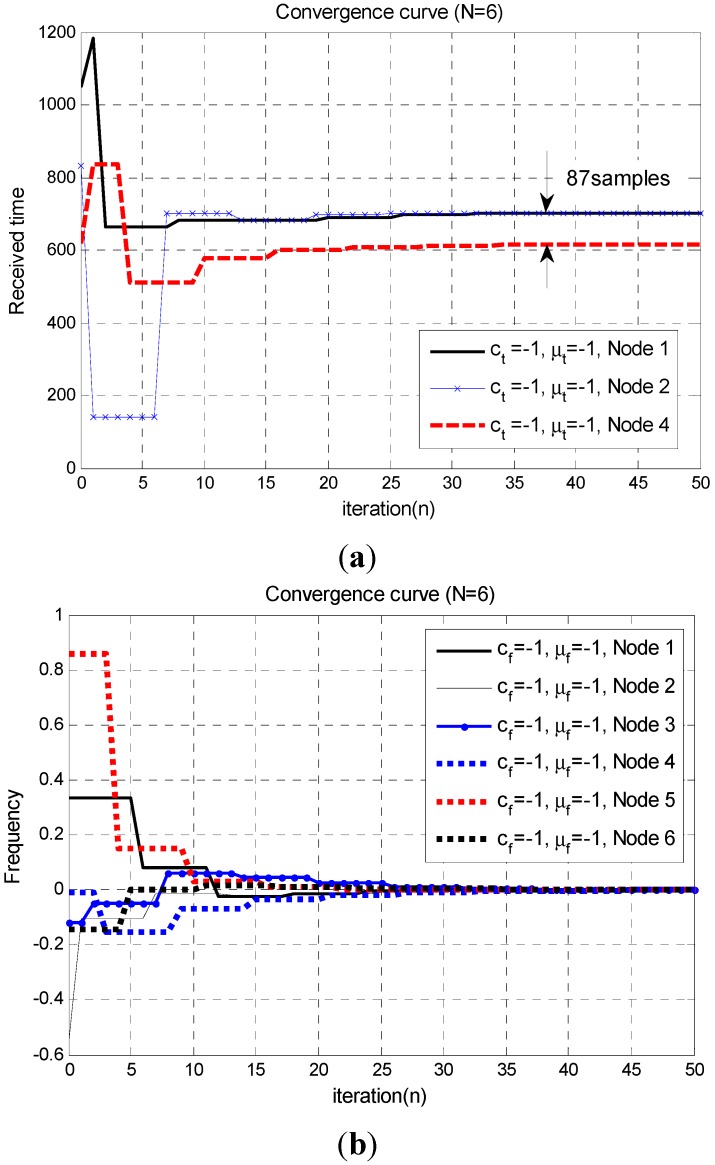
Convergence curve of the proposed synchronization technique in the multi-hop mesh network ( $\left[\tau_{12},\tau_{13},\tau_{23},\tau_{34},\tau_{45},\tau_{56},\tau_{46}]=[432,832,1048,618,281,296,149\right]$ ). (**a**) Convergence curve of the proposed time-synchronization technique (at Node 3); (**b**) Convergence curve of the proposed frequency-synchronization technique.

To evaluate the performance of the proposed synchronization technique in multi-hop mesh networks, a simulation is performed for the scenario shown in Figure 6. As shown in this figure, the maximum number of 1-hop neighbor nodes is three. The locations (propagation delays) of the nodes are randomly chosen. Figure 7 shows the convergence curves of the proposed synchronization algorithm in the multi-hop mesh network.

Figure 7a shows the convergence curves of the proposed time-synchronization algorithm when the propagation delays are present. This figure shows the convergence curves for 1-hop nodes, measured at Node 3. Here, we observe that the maximum TDoA between the nodes after the convergence is smaller than the CP length. Figure 7b shows the convergence curves of the proposed frequency-synchronization algorithm. In this figure, the convergence curves for all the nodes are shown because the frequency-synchronization algorithm is not affected by the propagation delays. Table 3 summarizes the synchronization success probability of the proposed technique in the multi-hop mesh network. Here, we observe that the synchronization success probability obtained by the distributed synchronization only is 98.88%.

**Table 3:** Synchronization success probability of the proposed technique in the multi-hop mesh network.

Step	Node 1	Node 2	Node 3	Node 4	Node 5	Node 6	Total
Only distributed synchronization	98.60%	98.60%	100%	98.70%	98.70%	98.70%	98.88%
Synchronization & self-organization	100%	100%	100%	100%	100%	100%	100%

## 5. Conclusions

In this paper, it was shown that the modified C-S model (discrete-time version of the flocking algorithm) cannot be directly applied for distributed time-synchronization in an OFDMA-based WMN with a TDoA, owing to the error-accumulation effect. By considering the propagation delays between the nodes, the proposed algorithm achieves time and frequency synchronization in a distributed manner using only the received signals from neighbor nodes. The proposed technique was shown to achieve a fast synchronization using a simple algorithm because it does not require any precompensation processes for the propagation delays through feedback channels. The information obtained from the self-organization step can be effectively used to construct 1-hop neighbor nodes in an OFDMA-based WMN, especially with a large number of nodes.
